# Global gene expression profile under low-temperature conditions in the brain of the grass carp (*Ctenopharyngodon idellus*)

**DOI:** 10.1371/journal.pone.0239730

**Published:** 2020-09-25

**Authors:** Mijuan Shi, Qiangxiang Zhang, Yongming Li, Wanting Zhang, Lanjie Liao, Yingyin Cheng, Yanxin Jiang, Xiaoli Huang, You Duan, Lei Xia, Weidong Ye, Yaping Wang, Xiao-Qin Xia

**Affiliations:** 1 Institute of Hydrobiology, Chinese Academy of Sciences, Wuhan, China; 2 The Innovative Academy of Seed Design, Chinese Academy of Sciences, Beijing, China; 3 University of Chinese Academy of Sciences, Beijing, China; 4 State Key Laboratory of Freshwater Ecology and Biotechnology, Institute of Hydrobiology, Chinese Academy of Sciences, Wuhan, China; National Cheng Kung University, TAIWAN

## Abstract

Grass carp is an important commercial fish widely cultivated in China. A wide range of temperatures, particularly extremely low temperatures, have dramatic effects on the aquaculture of this teleost. However, relatively few studies have characterized the molecular responses of grass carp exposed to acute cooling in natural environment. Here, we investigated the transcriptome profiles of the grass carp brain in response to cooling. Through regulation pattern analyses, we identified 2,513 differentially expressed genes (DEGs) that responded to moderate cold stress (12°C), while 99 DEGs were induced by severe low temperature (4°C).The pathway analyses revealed that the DEGs sensitive to moderate cold were largely enriched in steroid biosynthesis, spliceosome, translation, protein metabolism, phagosome, gap junction and estrogen signaling pathways. Additionally, we discerned genes most likely involved in low temperature tolerance, of which the MAPK signaling pathway was dominantly enriched. Further examination and characterization of the candidate genes may help to elucidate the mechanisms underpinning extreme plasticity to severe cold stress in grass carp.

## Introduction

As the “abiotic master factor” [1], water temperature controls all the physiological and behavioral parameters of fish [2]. Those fishes that live in low temperature environments throughout the year have evolved some unique physiological mechanisms to adapt to extremely cold conditions [3, 4]. It is evident that production of antifreeze protein, lack of hemoglobin and synthesis of tubulin can improve the adaptability of Antarctic fishes to low temperature environments [5–8]. However, eurythermal freshwater fishes are exposed to a wide range of fluctuations in water temperature, rather than in a relatively stable temperature environment. Therefore, it is crucial for eurythermic fish to adjust physiological processes quickly to respond to the acute cooling when severe cold fronts approach in winter.

Although extensive researches have been carried out to study the steady-state changes of fish after prolonged cold acclimation, such as the anion transport by mitochondrion-rich chloride cells in fish gills [9], proteome of the heart [10], changes of free amino acids in blood, and the hormone secretion in the brain [11, 12], we still know very little information about the physiological and molecular changes of fish in the early stages of cold acclimation. Pre-exposure of zebrafish larvae to moderate cold can significantly increase the survival rates under severe low temperature [13]. The fact indicates that earlier adjustment of fish in response to moderate low temperature is important for the tolerance of later severe cold stress. As an important concept in stress biology, allostasis has been described as the process of maintaining homeostasis under environmental stress by using dynamic physiological mechanisms of organisms [14–16]. Understanding the underlying mechanism of molecular changes during temperature changes can provide better insights into the extremely high plastic phenotypes of eurythermic fishes in response to cold stress.

As an eurythermic teleost widely distributed in the world, grass carp (*Ctenopharyngodon idellus*) can tolerate large changes in temperature. Despite the well-known phenotype that grass carp can quickly acquire tolerance to severe cold after acute cooling, the underlying mechanism remains unclear. Considering the essential role of the brain plays in processing environmental information and regulating homeostasis, the adaptive responses to cold stress in brain might be a key to understand the low temperature adaptability of the grass carp. Unfortunately, few studies have been conducted to address the molecular changes in the brain of grass carp exposed to cold stress. This study aims to identify the transcriptional responses in grass carp brain by profiling the global gene expression during cold acclimation.

## Materials and methods

### Fish and cold exposure

Fifty 14-month-old (approximately 100 g each) grass carps were collected from our research base at Wuhan, Hubei province. These grass carps were temporarily raised at 27±1°C in a 300 L tank for 2 weeks and fed 3 times a day. In order to simulate the cooling process in nature and eliminate the impact of cold shock on fish stress response, the water temperature was orderly adjusted from 27°C to 12°C, 4°C, 12°C, and finally 27°C at a rate of 0.25°C/h and a maximum 6°C/day. At each point, the temperature was maintained for 24 h, then the brains of eight randomly selected grass carps were obtained and ground in liquid nitrogen. Subsequently, 100 mg of ground tissue was added to 1 ml of TRIzol reagent and stored at -80°C. The experimental samples used for RT-qPCR were treated with the same project in the preparation of RNA-seq samples. At each temperature point, 3 fish were randomly selected, and the total brain RNA of each fish was separately obtained for RT-qPCR verification. The use of all experimental grass carp was approved by the Animal Research and Ethics Committees of the Institute of Hydrobiology, Chinese Academy of Sciences.

### RNA extraction and sequencing

Total RNA was extracted using TRIzol according to a standard protocol (Life Tech, USA). After quality inspection was performed, mRNA was enriched using oligo(dT) magnetic beads and fragmented with ultrasound. The double strand cDNA was synthesized using the mRNA fragments as template and was purified. Finally, sequencing adapters were ligated to the cDNA ends. The desired fragments were purified and enriched by 10-cycle PCR amplification. The library quality was checked by a Bioanalyzer 2100 (Agilent). The qualified library products were used for single-ended sequencing via Illumina Hiseq TM2000. The accession number of the raw data is PRJCA001614 (BIG: http://bigd.big.ac.cn/gsa).

### Data processing and basic statistical analysis

NGSQCToolkit (version 2.3.3) [17]was used to eliminate non-useful data with default parameters. The remaining clean reads were mapped to the reference genome [18] by Tophat2 (version 2.0.7) [19], and the transcripts were assembled with StringTie (version v1.3.1c) [20]. The sequences were from the Blast to nr database, and those with an e-value ≤1e-5 were annotated. The read counts and Transcripts Per Kilobase Million (TPM) were calculated by salmon (version 0.12.0) [21] with a nonalignment algorithm. The default parameters were used in all software above. The results above are presented in supplementary tables (S1, S2 and S3 Tables).

### Identification of Differentially Expressed Genes (DEGs) and enrichment analysis of pathways

edgeR [22] was used for differential expression analysis of genes through the generalized linear model (glm) approach with likelihood ratio tests [23]. The genes that were stably expressed with a coefficient of variation of less than 0.2 during the whole process were used to estimate the variance of samples and to identify DEGs. The criteria to define differential expression were false discovery rate (FDR) ≤ 0.01 and absolute log2 of TPM ratio ≥ 1. The pathways database was downloaded with the API interface provided by KEGG. A total of 318 pathway maps, of which the ratio of grass carp genes annotated to all genes of the map was more than 20%, were selected for the enrichment analysis. The statistical test for enrichment analysis was performed by R scripts executing Fisher’s exact test with the *p* value ≤ 0.05.

### Validation of expression profiles using RT-qPCR

Given the instability of beta-actin expression under cold conditions, the gene RPL13A was used as the internal control, which was steadily expressed during cold adaptation [24]. Then, 4 genes (5 transcripts) were selected to validate, and the length of the PCR products was approximately 120–250 bp (Primer list in S4 Table) and Tm ~55°C.

## Results

### Transcriptome assembly and statistics

We conducted a project that decreased the water temperature from 27°C to 4°C and increased it back to 27°C to simulate the temperature changes in nature. The RNA-seq data sets at five temperature points (27°C -> 12°C -> 4°C -> 12°C -> 27°C) that sequentially reached during the process were obtained and named A, B, C, D, E. Each data set had 8.45±0.26 M clean reads with a 96.03±0.76% mapping rate (S1 Table). Subsequently, we implemented the transcriptome assembly using published gene structure annotation information of grass carp as a reference [18] and generated a new annotation file containing 53,288 transcripts of 37,756 genes.

### Differential expression of genes

The genes assembled above with a abundance > 5 TPM in either control or treatment samples were regarded as being transcribed and a total of 11,019 expressed genes were identified in our research (S2 and S3 Tables). Differentially expressed genes (DEGs) can be obtained by comparing the transcriptome profile of any treated group (B, C, and D) to the control group (A), or by comparing every two adjacent temperature points. For convenience, we named them as control-DEGs and adjacnet-DEGs respectively. Considering the fact that the DEGs between B and A can be counted in both types, there are four comparisons for control-DEGs (B vs. A, C vs. A, D vs. A, and E vs. A) and four for adjacent-DEGs (B vs. A, C vs. B, D vs. C, and E vs. D). A total of 2,643 DEGs were detected by all the seven comparisons.

**Fig 1:**
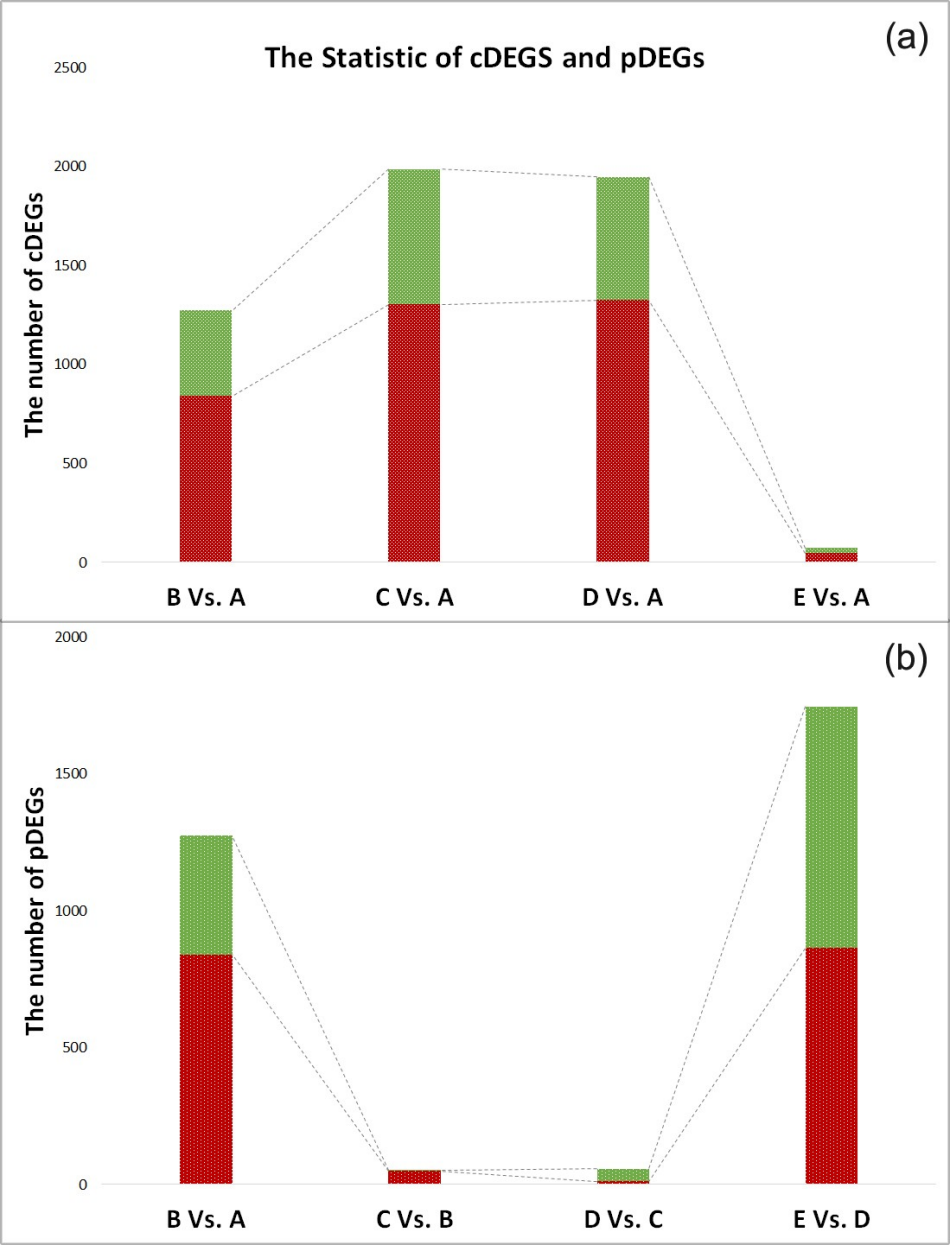
Stacked bar of DEGs. a. The genes differentially expressed (DEGs) in group B (12°C), group C (4°C), group D (12°C) and group E (27°C) in comparison to control group A (27°C). b. The differentially expressed genes between two adjacent groups. In both plots, red represents upregulated genes, and green represents downregulated genes.

To identify the genes involved in the cold stress response, control-DEGs were discerned in terms of relative abundance (fold change > 2, *p* value <0.01). As a result, 840 genes were upregulated and 433 were downregulated in the group B, while 1,304 genes were upregulated and 683 were downregulated in the group C. Additionally, 1,326 genes were upregulated and 622 downregulated in group D, while up- and down-regulated genes were respectively 47 and 26 in group E (Fig 1A and S5 Table).

In order to further investigate the genes closely associated with severe cold stress, we estimated the differences between profiles of moderate and severe cold treatments using adjacent-DEGs. In the four comparisons, there were as many as 1,273 and 1,745 adjacent-DEGs during the cooling/heating process between 27°C and 12°C, while only 52 and 58 DEGs were identified between 12°C and 4°C (Fig 1B and S5 Table).

### Expression patterns of DEGs

**Fig 2:**
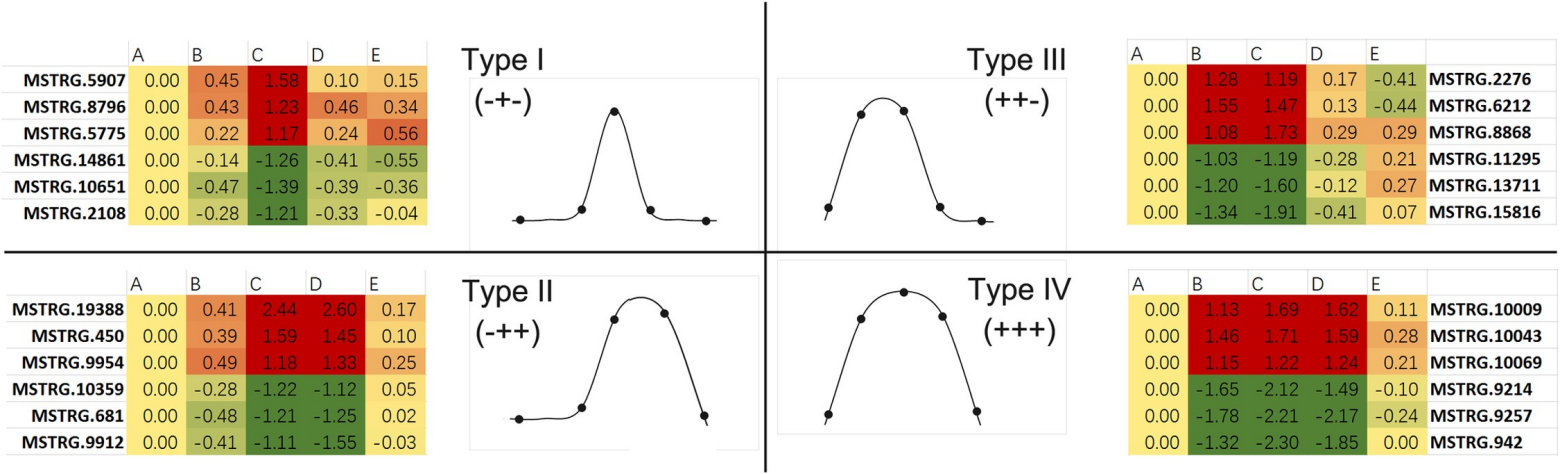
Schematic of gene expression patterns. The log2 (fold change) of gene expression in test group B (12°C), group C (4°C) and group D (12°C) compared with control group A (27°C) are showed in the tables in which green represents downregulation and red represents upregulation. Moreover, significant up- or down-regulation is marked as positive (+), and non-significant expression is labeled as negative (-). The curves display the differential expression of genes in group A, B, C, D, E.

From the control-DEGs, we identified four expression patterns in response to temperature variation (S6 Table). Given that "+" represents a significant gene regulation (up or down) and "-" represents no regulation at each time point (B, C and D), the four patterns can be depicted as type I (-+-), type II (-++), type III (++-), and type IV (+++), and the included DEGs were 40, 59, 182 and 2,331 correspondingly (Fig 2). The changes in transcription level suggest that type I and type II cover genes responding to severe cold temperature while genes of type III and type IV might play roles in moderate cold stress.

### Pathway analysis of DEGs

**Fig 3:**
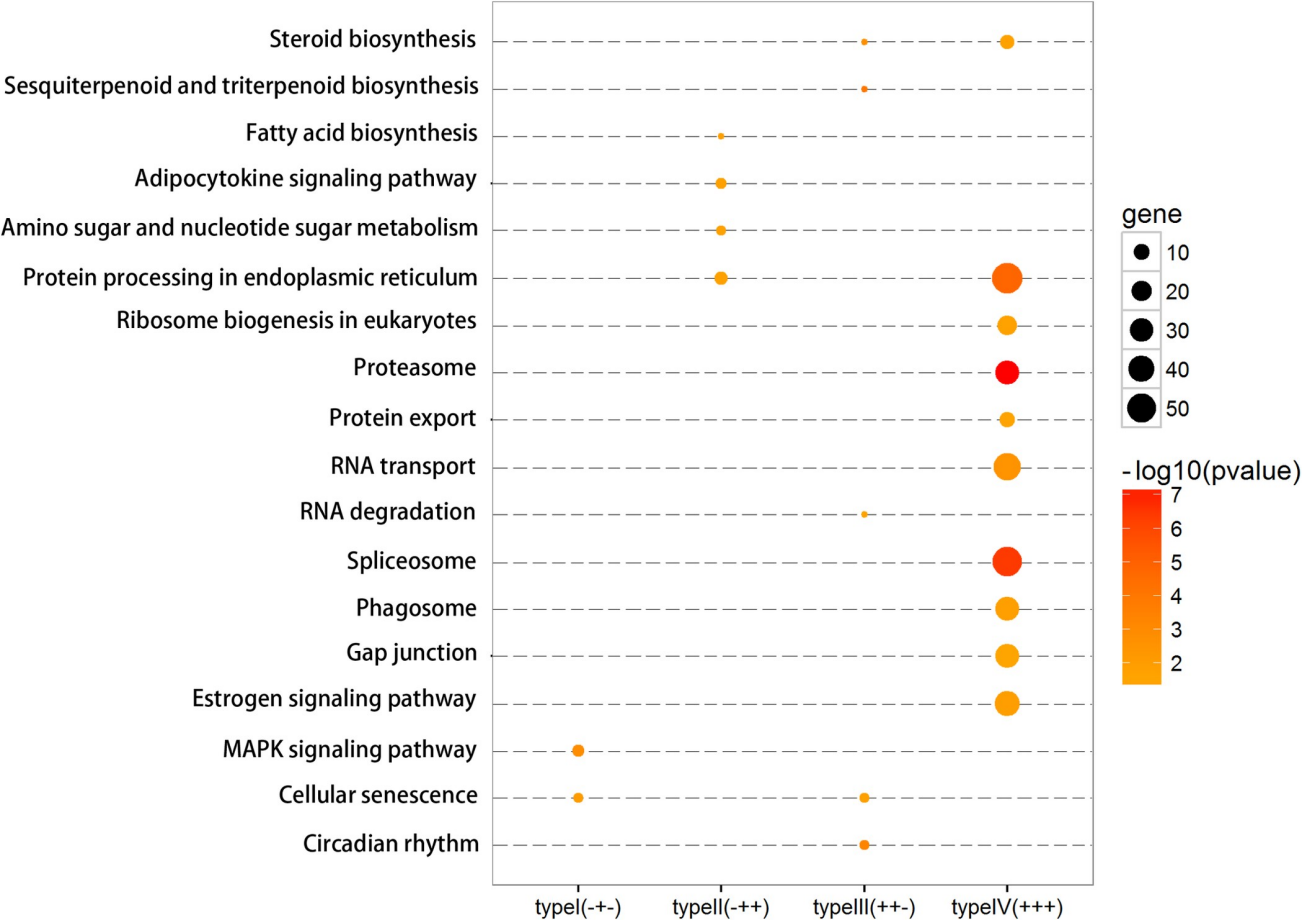
Pathway enrichment of DEGs. Pathways enriched from DEGs are displayed according to gene expression patterns.

Through KEGG analyses, the DEGs with one of the four expression patterns were significantly enriched in 18 pathways related to metabolism, transcription, translation, signal transduction and cellular processes (Fig 3). Specifically, type I genes were mostly enriched in the MAPK signaling pathway; type II genes covered 4 pathways for protein processing in endoplasmic reticulum, amino sugar and nucleotide sugar metabolism, adipocytokine and fatty acid biosynthesis; type III genes were involved in steroid biosynthesis, RNA degradation, circadian rhythm and cellular senescence; type IV genes dispersed in 10 pathways associated with steroid biosynthesis, spliceosome, translation, protein metabolism, phagosome and estrogen signaling pathway.

### Validation of RNA-seq data with RT-qPCR

**Fig 4:**
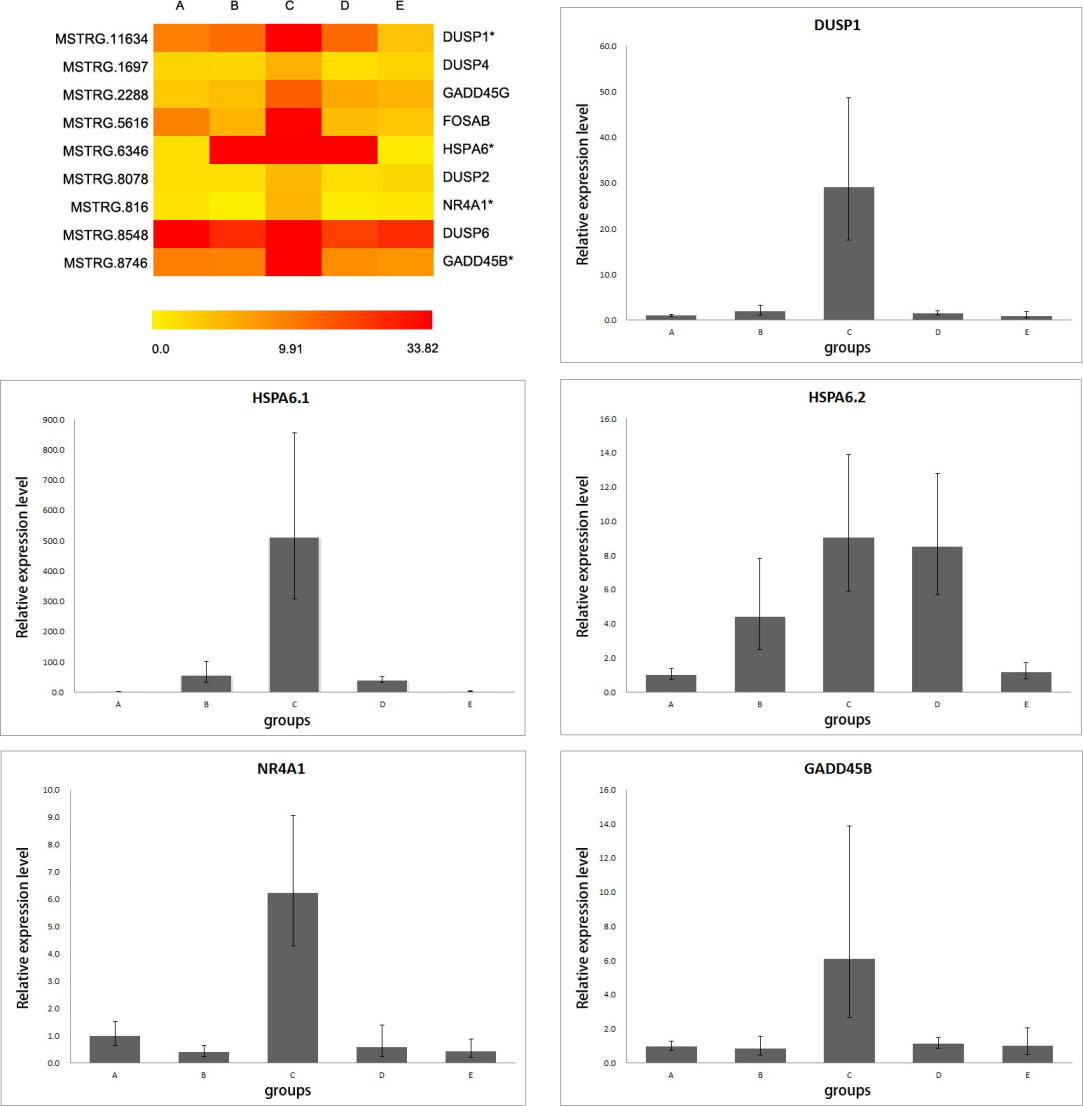
Comparison of RNA-seq profiles with RT-qPCR data. a. Heatmap of differentially expressed genes with TPM in RNA-seq data. b1-b5. Expression profiles of DUSP1, HSPA6 (HSPA6.1, HSPA6.2), NR4A1 and GADD45B with the RT-qPCR results.

Considering the significance of the MAPK signaling pathway in the cold response, four extensively highly expressed genes (DUSP1, HSPA6, NR4A1 and GADD45B) at 4°C were verified by real-time quantitative PCR (RT-qPCR). Among these genes, two isoforms of HSPA6 (HSPA6.1 and HSPA6.2) were validated separately. The results of RT-qPCR were almost identical to those of RNA-seq (Fig 4).

## Discussion

To identify genes involved in earlier stage of cold acclimation, we analyzed the transcriptomes in the brain of grass carp subjected to graded chronic cooling and heating regime (27°C -> 12°C -> 4°C -> 12°C -> 27°C). In general, much more DEGs (1,273) were detected during the first cooling process (27°C -> 12°C) in contrast to 52 adjacent-DEGs in the second cooling (12°C -> 4°C, S5 Table). These data may indicate a fact that the switch from normal growth temperature to moderate cold has a huge impact on grass carp, and drive the grass carp to complete most necessary physiological adjustments to accommodate the cold environment so that only minor genes closely relevant to severe cold tolerance needed to regulate for the further cooling to severe cold. However, we noticed that more genes involved in the two cooling processes. Ignoring the recurrence of a few genes, we got 1,325 DEGs by simply summing up the two cooling steps (1,273 in B vs. A and 52 in C vs. B). In fact, there were a total of 1,987 DEGs in C vs. A. Numerically, 662 (1,987–1,325) additional DEGs were found in the comparison C vs. A. Since these additional genes were not detected in B vs. A nor in C vs. B, most likely their expression changed very slightly in each step, and eventually accumulated to a significant level after cooling to 4°C. Interestingly, there were few transcriptional changes induced by raising temperature from 4°C to 12°C, whereas the expression level of almost all DEGs were restored to normal extent with temperature back to 27°C, it is roughly commensurate with the gene expressions when the temperature dropped. Such a symmetry confirms that the expressions of certain genes are temperature-dependent and subjected to specific temperature extent.

In order to examine the temperature-dependent expression of genes in cold acclimation, we analyzed the patterns of gene expression and classified the control-DEGs into four expression types in accordance with the relative abundance of transcripts in group B, C, D (12°C -> 4°C -> 12°C) as compared to the gene expression profiles in control group A (27°C). Additionally, according to the sensitivity of gene transcription in response to moderate cold (12°C), we suggested control-DEGs with expression type I (-+-) or type II (-++) were likely to participate in responses to severe cold tolerance, and control-DEGs regulated as type III (++-) or type IV (+++) pattern were closely associated with moderate cold stress. Given that genes with common expression pattern are likely to have coordinate functions, pathway analyses were conducted upon DEGs with coherent transcriptional pattern to better understand overall patterns of biological processes involved in cold acclimation.

### Responses to moderate cold stress

The largest proportions of DEGs were regulated as pattern type III (++-) and type IV (+++), of which genes were consistently overrepresented in group B (12°C) and group C (4°C) compared with control group A (27°C). This indicates that many biological processes are likely to be regulated under moderate cold stress (12°C) and remain stable until the temperature rises. Consequently, the estimation by the pathway analysis on these DEGs gave an insight into the brain's responses to moderate cold stress. In addition, considering the significance indicated by our results and prevalence in previous studies on cold acclimation, we paid extra attention to pathways associated with lipid metabolism and alternative splicing.

#### Lipid metabolism

As an adaptive response of poikilothermic organisms in the cold, homeoviscous adaptation has been extensively studied, especially with regard to the composition of membrane lipids [25]. Although the most commonly observed alterations of membrane lipids involve changes in the unsaturation of fatty acids bound to phospholipids, it has been demonstrated that changes in the ratio of phospholipid to cholesterol participate in homeoviscous adaptation [26]. Recent studies have shown that cholesterol synthesis is probably involved in the cold acclimation of common carp [27] and yellow drum [28].

**Fig 5:**
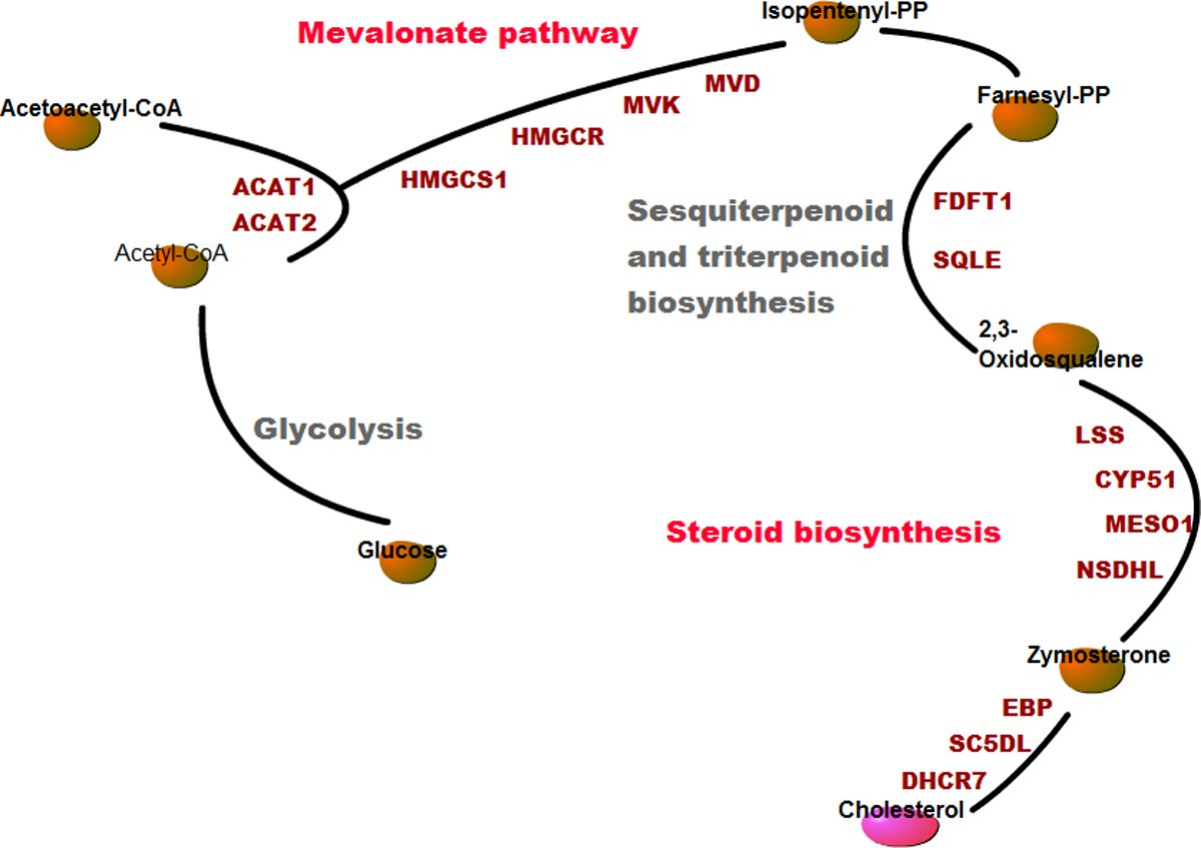
Schematic diagram of the cholesterol synthesis pathway.

Consistent with previous studies, the terpenoid backbone biosynthesis pathway and steroid biosynthesis pathway were significantly enriched in grass carp brain exposed to cold stress. Six genes (HMGCR, HMGCS1, ACAT2, MVD, IDI1 and MVK) were upregulated at 12°C and 4°C in terpenoid backbone biosynthesis pathway. At the same time, genes associated with the steroid biosynthesis pathway (FDFT1, SQLEA, LSS, MSMO1, NSDHL, EBP, SC5D and DHCR7) were also remarkably upregulated at 12°C and 4°C. Interestingly, the terpenoid backbone biosynthesis pathway is located upstream of the steroid biosynthesis pathway. Combining these two pathways, we clearly observed an unimpeded route to transform acetyl-CoA to cholesterol (Fig 5). Considering the effect of cholesterol on manipulating the fluidity and flexibility of cell membranes at low temperatures, we suggest that the cholesterol may play an important role in cold stress in the brain of grass carp. Furthermore, according to the overall pattern of differential gene expression in the cholesterol biosynthesis pathway, no matter the temperature changes from 4°C to 12°C, cholesterol concentration might increase under moderate cold stress and persist throughout the whole cold period, which supports the idea that the regulation of cholesterol concentration may be a fundamental part of responses to cold in grass carp brain.

#### Alternative splicing

As an important post-transcriptional modification mechanism, alternative splicing can produce different transcripts from the same pre-mRNA. Its role in the cold resistance of plants has been studied [29, 30], and some researchers have noticed the effect of this mechanism on the cold adaptation of fish. There is evidence that alternatively spliced isoforms of delta 9-acyl-CoA desaturase has a significant response to cold in common carp [31], while RNA-seq analysis found that cold stress of laval zebrafish can regulate the alternative splicing of 197 genes [32]. Moreover, comparative investigations on the extent of gene alternative splicing in Atlantic killifish, threespine stickleback and zebrafish indicated that this mechanism is common in fish responses to cold [33].

Similarly, we found that the genes related to the spliceosome were significantly upregulated at temperatures below 12°C, just like cold stress studies on zebrafish [32] and yellow drum [28]. In addition, although alternative splicing is specific in different fishes, there are 29 common genes found to be regulated by alternative splicing in Atlantic killifish, threespine stickleback and zebrafish in response to cold stress [33]. However, according to the differential expression pattern of each transcript isoform, only 14 of the 29 common genes were regarded as alternatively splicing in our research (S7 Table). This divergence is most likely due to the differences in the strategies applied by species or the tissue specificity.

The 70 kDa heat shock protein 6 gene (HSPA6) is the 6th member of heat shock protein family A (Hsp70), a famous molecular chaperone family. Members of Hsp70 are often active in cold acclimation or cold shock [34, 35]. In contrast to the overall expression pattern of HSPA6 assessed by RNA-seq assay, the two isoforms of HSPA6 presented different expression patterns in our RT-qPCR analysis. One isoform (HSPA6.1) was highly upregulated at 4°C, while the other isoform (HSPA6.2) was incrementally expressed with temperature cooling from 27°C to 4°C. Considering the dissimilar sensitivity of two isoforms to temperature, we hypothesize that the HSPA6 is modulated under the control of alternative splicing during the cold acclimation of grass carp. In conclusion, the study exhibited the possibility that alternative splicing participates in the cold acclimation of grass carp.

### Responses to severe cold tolerance

In contrast to those control-DEGs at 12°C, the genes which were differentially expressed only at 4°C were more related to severe cold stress. Accordingly, we focused on the functional interpretation of DEGs exclusively triggered at 4°C, of which 5 DEGs (DUSP1, DUSP2, GADD45B, GADD45G and CACNA1D) were enriched in the MAPK signaling pathway.

Although the MAPK signaling pathway is extensively involved in the cold acclimation of plants and animals [36–38], the involved genes vary in different species, indicating that distinct mechanisms are adopted by different organisms. Previous studies have shown that DUSP1 is induced in response to hypoxia [39] and acute thermal exposure [40]. There is also evidence that the suppression of DUSP1 can significantly increase the apoptotic rate of zebrafish ZF4 cells at low temperatures [41], and recent studies have revealed the link between DUSP1 and low temperature-induced embryonic diapause in blue-breasted quail [42]. based on these studies, we speculate that DUSP1 plays an pivotal role in regulating cell growth and apoptosis under cold stress.

Liebermann and Hoffman reviewed the effects of GADD45 proteins on cell death and survival under cell stress, and pointed out that GADD45A, GADD45B and GADD45G play different roles in stress signal transduction [43]. Although a recent study revealed that mice lacking GADD45G had defects in the thermogenic response to cold [44], we still know very little about the role of GADD45 in cold stress. According to our results, both GADD45B and GADD45G appeared to be involved in response to severe cold exposure, while the expression of GADD45A was not significantly different at low temperature, indicating that GADD45B and GADD45G rather than GADD45A participate in the response of grass carp to cold stress.

In addition, recent studies have shown that the repression of gene ARID1A lead to the upregulation of stress-sensors and apoptosis-regulators GADD45B and DUSP1 [45]. Intriguingly, we also detected a significant downregulation of ARID1A at 4°C, while GADD45B and DUSP1 were remarkably upregulated. Consequently, we assume that ARID1A is downregulated under cold stress, resulting in an increase of GADD45B and DUSP1, which are likely involved in cell growth and apoptosis.

## Supporting information

S1 Table Summary of RNA-seq data. (XLSX)

S2 Table Genes annotation. (XLSX)

S3 Table TPM of annotated genes. (XLSX)

S4 Table The primers sequences used for PT-qPCR. (XLSX)

S5 Table The statistics of DEGs/DETs. (XLSX)

S6 Table The expression pattern classification of DEGs. (XLSX)

S7 Table Alternatively Spliced (AS) genes commonly identified in four species. (XLSX)

S8 Table Statistics of the transcript classification. (XLSX)
